# Sepsis incidence, suspicion, prediction and mortality in emergency medical services: a cohort study related to the current international sepsis guideline

**DOI:** 10.1007/s15010-024-02181-5

**Published:** 2024-02-19

**Authors:** Silke Piedmont, Ludwig Goldhahn, Enno Swart, Bernt-Peter Robra, Carolin Fleischmann-Struzek, Rajan Somasundaram, Wolfgang Bauer

**Affiliations:** 1 Charité - Universitätsmedizin Berlin, Zentrale Notaufnahme Campus Benjamin Franklin, Berlin, Germany; 2 Institut für Sozialmedizin und Gesundheitssystemforschung, Otto-von-Guericke-Universität Magdeburg, Magdeburg, Germany; 3 Institut für Infektionsmedizin und Krankenhaushygiene, Universitätsklinikum Jena, Jena, Germany

**Keywords:** Sepsis, Incidence, Mortality, Emergency medical services, Paramedic, Screening

## Abstract

**Purpose:**

Sepsis suspicion by Emergency Medical Services (EMS) is associated with improved patient outcomes. This study assessed sepsis incidence and recognition by EMS and analyzed which of the screening tools recommended by the Surviving Sepsis Campaign best facilitates sepsis prediction.

**Methods:**

Retrospective cohort study of claims data from health insurances (*n* = 221,429 EMS cases), and paramedics’ and emergency physicians’ EMS documentation (*n* = 110,419); analyzed outcomes were: sepsis incidence and case fatality compared to stroke and myocardial infarction, the extent of documentation for screening-relevant variables and sepsis suspicion, tools’ intersections for screening positive in identical EMS cases and their predictive ability for an inpatient sepsis diagnosis.

**Results:**

Incidence of sepsis (1.6%) was similar to myocardial infarction (2.6%) and stroke (2.7%); however, 30-day case fatality rate was almost threefold higher (31.7% vs. 13.4%; 11.8%). Complete vital sign documentation was achieved in 8.2% of all cases. Paramedics never, emergency physicians rarely (0.1%) documented a sepsis suspicion, respectively septic shock. NEWS2 had the highest sensitivity (73.1%; Specificity:81.6%) compared to qSOFA (23.1%; Sp:96.6%), SIRS (28.2%; Sp:94.3%) and MEWS (48.7%; Sp:88.1%). Depending on the tool, 3.7% to 19.4% of all cases screened positive; only 0.8% in all tools simultaneously.

**Conclusion:**

Incidence and mortality underline the need for better sepsis awareness, documentation of vital signs and use of screening tools. Guidelines may omit MEWS and SIRS as recommendations for *prehospital* providers since they were inferior in all accuracy measures. Though no tool performed ideally, NEWS2 qualifies as the best tool to predict the highest proportion of septic patients and to rule out cases that are likely non-septic.

**Supplementary Information:**

The online version contains supplementary material available at 10.1007/s15010-024-02181-5.

## Introduction

Sepsis causes an estimated 20% of all global deaths [1]. There is a great potential to save lives and maintain patients’ quality of life by recognizing and treating sepsis earlier [2–4]. As most sepsis cases start outside of the hospital [5, 6], Emergency Medical Services’ (EMS) sepsis knowledge and screening is crucial: EMS’ sepsis suspicions are associated with shortened time to in-hospital treatment and reduced mortality risks [7–9]. Yet, EMS only recognize a minority of sepsis cases [10, 11].

To aid sepsis screening, the 2021 updated international guideline by the Surviving Sepsis Campaign [12] (SSC) mentions:

- Criteria for Systemic Inflammatory Response Syndrome (SIRS [13]),
- Modified Early Warning Score (MEWS [14]) and
- National Early Warning Score 2 (NEWS2 [15])
- quick Sequential [Sepsis-related] Organ Failure Assessment (qSOFA [16]).

However, its authors “*recommend against using qSOFA, compared with SIRS, NEWS, or MEWS as a single screening tool for sepsis or septic shock*” ([12], p. e1064). This leaves questions which tool to prefer or how to combine them.

In the past, the SSC guidelines have strongly influenced national screening recommendations (e.g., in Germany and Japan [17, 18]). Yet, the current SSC guideline’s evidence seems improvable as it does not cite a single study which compares all four screening tools’ predictive ability for sepsis and mostly relates to studies on the prediction of mortality—not sepsis. Furthermore, its recommendations base on hospital studies only (cf. [12]). Since screening tools’ predictive ability depend on the setting they are applied in and two out of four screening tools are not completely feasible for EMS (cf. [19]), this leaves questions on their usefulness in the prehospital setting. Furthermore, national sepsis or EMS guidelines often recommend only one specific tool, e.g., the qSOFA in Germany or NEWS2 in England [17, 20, 21], or tools differ by region or EMS providers (e.g., with some Swedish EMS using NEWS2) [22]. All of these create uncertainty which screening tool is best in the EMS setting.

Improvable assessment and documentation rates for vital signs as well as general sepsis knowledge are additional challenges hindering early sepsis recognition [8, 23–27]. To inform EMS about the relevance of sepsis screening, the current study also compares the sepsis incidence and sepsis-related case fatality to those of myocardial infarction or stroke.

## Methods

### Aim

The study answers the following questions:

- *Incidence and case fatality*: How do sepsis incidence and case fatality compare to those of myocardial infarction and stroke?
- *Documentation of screening-relevant parameters*: How complete is documentation of screening-relevant parameters (e.g., temperature)? How do documentation rates differ between paramedics versus prehospital emergency physicians or between patients with versus without sepsis?
- *Sepsis suspicion*: How often do EMS document a sepsis suspicion? How often would EMS cases screen sepsis-positive, respectively with sepsis suspicion, if EMS staff had applied screening tools?
- *Comparison of screening tools*: How is each screening tool’s predictive ability for sepsis? How frequently do tools label different patients with sepsis suspicion?

### Data sources

**Fig. 1:**
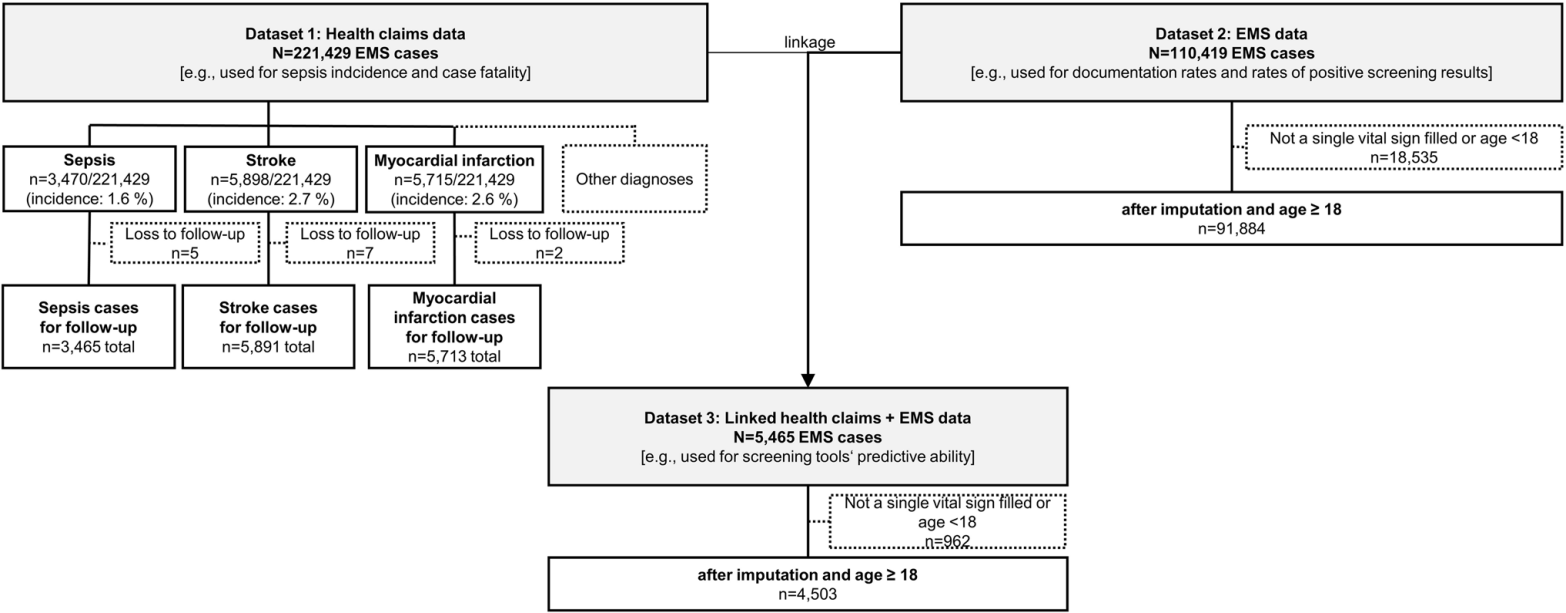
Sample sizes for individual analyses on case level (Dataset #3: To be linkable, EMS cases had to be billed by respective health insurance company [dataset #1] and conducted by respective EMS provider [dataset #2])

The retrospective cohort study based on claims data by ten health insurance companies, plus EMS documentation by paramedics (PM) and emergency physicians (EP) from Germany (Fig. 1). Linking the pseudonymized data allowed determination of the screening tools’ predictive ability during EMS care for the outcome of an inpatient sepsis diagnosis following EMS care (linkage details: [28]).

- *Dataset #1 (health claims data)*: 221,429 German-wide EMS cases billed by 10 participating health insurance companies, with ground and aerial vehicles indicating emergencies in the year 2016 (including individual follow-up until December 31, 2017, for diagnosis and case fatality);
- *Dataset #2 (EMS data)*: 110,419 EMS cases documented by PM (*n* = 106,936) and EP (*n* = 3483) in the year 2016 in the federal states Bavaria and Baden-Württemberg, independent of any certain health insurance company (details in [28]); dataset includes EMS’ vital signs documentation and sepsis suspicions
- *Dataset #3 (health claims #1 linked with EMS data #2)*: 5465 linkable EMS cases

Datasets #1 to #3 contained *all* EMS cases, e.g., cases resulting in in- or outpatient care, with or without conveyance and death on-site (see Online Resource 1 for rate of inpatient admissions).

### Sepsis incidence and case fatality

Based on the health claims data (dataset #1), the study assessed sepsis incidence, hospital and 30-day case fatality rate for all EMS cases. A case was considered to result in an inpatient sepsis diagnosis if the diagnosis belonged to a hospital stay starting on the day of EMS use. A case without an inpatient sepsis diagnosis was considered to be non-septic. The diagnosis relied on an operationalization for German statutory health claims data to detect severe sepsis and septic shock ([29]; Online Resource 2). This strategy takes into account the current sepsis definition, which requires one or more organ dysfunctions [12]. Sepsis incidence and case fatality were compared to the inpatient diagnoses of myocardial infarction (ICD codes I21, I22) and stroke (ICD I63, I64).

### Sepsis-relevant documentation, suspicion, and screening

In the EMS documentations (dataset #2), screening-relevant parameters (e.g., temperature) were deemed filled, if either the first or second assessment during EMS care was documented. Medically implausible data were transformed to missing values (Online Resource 3).

EMS’ sepsis suspicions based on the standardized codes of the “Minimale Notfalldatensatz” (translated: “minimal emergency dataset”). Additionally, one region provided free text fields for preliminary diagnoses by paramedics (Online Resource 2).

Based on the recommended thresholds, retrospectively calculated qSOFA and SIRS scores ≥ 2, MEWS score ≥ 4 and NEWS2 scores ≥ 5 were judged as a positive screening result. We report the tools’ predictive ability for an inpatient sepsis diagnosis (the “gold standard”) of patients ≥ 18 years. As information on urine output, paCO_2_, and leukocyte count is not routinely available to EMS staff, those Modified Early Warning Score (MEWS) or SIRS variables were omitted (Online Resource 4). In cases of missing values for screening-relevant variables, those values were imputed (Online Resource 3). To allow comparability with other studies, we also report screening results using other methods of handling missing values in Online Resource 5.

### Analysis

Descriptive and interferential analyses were carried out using IBM® SPSS® Statistics Version 26 and Microsoft Excel. Venn diagrams were developed using RStudio (Version 4.0.2) and PowerPoint (Version 2309). All analyses used pairwise deletion except when reporting how frequent sepsis-relevant variables were (un)documented. Independent samples were compared with Pearson’s (cf. Table 1), dependent samples with McNemar’s chi^2^ tests (for comparisons of screening results), accepting an α ≤ 0.05 without correction for multiple testing. In an additional sensitivity analysis, conclusions were not affected by Bonferroni correction for multiple testing.

**Table 1:** Completeness rates for variables relevant for sepsis screening

	EMS Dataset #2 (*n* = 110,419); differentiated by EMS staff					linked Dataset #3 (*n* = 5465); differentiated by resulting inpatient sepsis diagnosis				
	All	Emergency physicians (*n* = 3483)	Paramedics (*n* = 106,936)	*p*-Value	Cramer-V	All	With sepsis (*n* = 87)	Without sepsis (*n* = 5378)	*p*-Value	Cramer-V
Temperature^b,c^^,d^	17.8% [17.6; 18.0]	19.8% [18.5; 21.2]	17.7% [17.5; 18.0]	< 0.01	0.01	21.8% [20.8; 23.0]	46.0% [35.8; 56.4]	21.5% [20.4; 22.6]	< 0.01	0.07
Respiratory rate^a,b^^,c,d^	27.9% [27.6; 28.1]	53.3% [51.6; 54.9]	27.0% [26.8; 27.3]	< 0.01	0.10	32.5% [31.2; 33.7]	40.2% [30.4; 59.7]	32.4% [31.1; 33.6]	0.12	0.21
Glasgow Coma Scale^a^	77.7% [77.5; 78.0]	67.8% [66.2; 69.3]	78.1% [77.8; 78.3]	< 0.01	0.04	80.3% [79.2; 81.3]	80.5% [71.2; 87.7]	80.3% [79.2; 81.4]	0.7	< 0.01
Oxygen saturation^d^	79.2% [79.0; 79.5]	85.4% [84.2; 86.5]	79.0% [78.8; 79.3]	< 0.01	0.03	80.5% [79.4; 81.5]	86.2% [77.8; 92.2]	80.4% [79.3; 81.4]	0.18	0.02
Systolic blood pressure^a,b^^,d^	79.0% [78.8; 79.3]	86.9% [85.7; 88.0]	78.8% [78.5; 79.0]	< 0.01	0.04	78.9% [77.8; 80.0]	85.1% [76.5; 91.4]	78.8% [77.7; 79.9]	0.16	0.02
Heart rate^b,c^^,d^	81.7% [81.5; 82.0]	88.7% [87.6; 89.7]	81.5% [81.3; 81.7]	< 0.01	0.03	82.5% [81.5; 83.5]	86.2% [77.8; 92.2]	82.5% [81.5; 83.5]	0.36	0.01
Consciousness^b,d^	86.3% [86.1; 86.5]	68.6% [67.0; 70.1]	86.9% [86.7; 87.1]	< 0.01	0.09	88.0% [87.1; 88.8]	89.7% [82.0; 94.8]	88.0% [87.1; 88.8]	0.63	0.01

95% confidence intervals in square brackets

^a^Part of qSOFA

^b^Part of MEWS

^c^Part of SIRS

^d^Part of NEWS2

## Results

Out of 221,429 EMS cases, 3470 resulted in an inpatient sepsis diagnosis (dataset #1; Fig. 1). Those patients tended to be older and more frequently male compared to non-septic cases (Online Resource 1, incl. prevalence).

### Sepsis incidence and case fatality

**Fig. 2:**
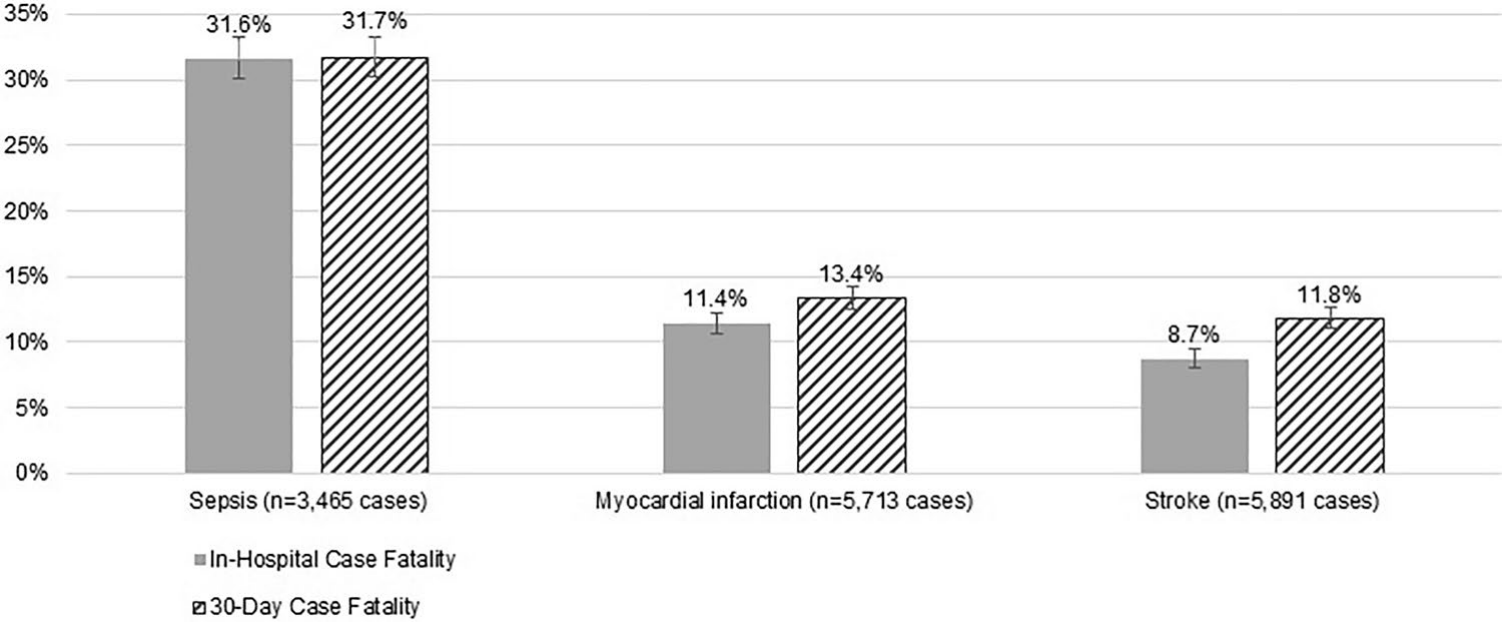
In-hospital and 30-day case fatality for inpatient sepsis, myocardial infarction and stroke following EMS use (Dataset #1)

Sepsis incidence of 1.6% [1.5;1.6%] was slightly lower than the incidence of 2.7% [2.6; 2.7%] for stroke and 2.6% [2.5; 2.6%] for myocardial infarction (dataset #1; *n* = 221,429). The hospital and the 30-day case fatality for sepsis were significantly higher, with 31.6% and 31.7%, respectively, for sepsis and 13.4% or lower for myocardial infarction or stroke (Fig. 2; Online Resource 1). Within 30 days, 1095 sepsis, 697 stroke and 651 myocardial infarction cases died.

Key result: Sepsis was about three times more likely to be fatal compared to myocardial infarction or stroke

### Sepsis-relevant documentation and suspicion

Within dataset #2, paramedics (PM) never checked sepsis suspicion as “yes” (0 out of *n* = 106,936 PM cases). Analysis of free texts fields also revealed no documentation of any sepsis suspicions. Prehospital emergency physicians (EP) documented a suspicion of “septic shock” in 0.1% [0.02; 0.3%] of their cases (5 out of *n* = 3,483).

Only in 8.2% [8.0; 8.4%] of all cases (9053 out of *n* = 110,419), *all* parameters listed in Table 1 were documented. Temperature was the least often recorded. Completeness rates for screening-relevant variables are similar for both types of staff (PM versus EP; negligible to weak associations of Cramer-V ≤ 0,1 for the relationship between completeness rates and type of staff). In the linked dataset #3 (*n* = 5465), completeness rates were similar for most screening-relevant variables in cases of those with versus without sepsis (Table 1); only temperature was documented significantly more often for cases resulting in sepsis, but the effect size was low (46.0% vs. 21.5%; *V* = 0.07).

Key results: Paramedics’ and prehospital Emergency Physicians’ documentations of vital signs and sepsis suspicions are improvable, also for patients diagnosed as septic in the hospital

### Predictive ability of sepsis screening tools

Considering the “abnormal” vital signs in dataset #2 and #3, EMS could have labeled more patients with sepsis suspicions, if they had applied screening tools: The percentage of cases which would have screened positive (i.e., with sepsis suspicion) during EMS care was lowest for qSOFA and highest for NEWS2 (Table 2). qSOFA had the highest specificity and positive predictive value, while NEWS2 had the highest sensitivity and AUROC. SIRS and MEWS were inferior in all accuracy measures.

**Table 2:** Screening results with qSOFA, MEWS, SIRS and NEWS2 for all cases with age ≥ 18 years

**Imputed EMS dataset #2; *n*** **= 91,884; unknown number of cases with inpatient sepsis**				
	qSOFA	MEWS	SIRS	NEWS2
% of cases labeled with sepsis suspicion by respective tool [95% CI]	4.3%	13.9%	6.0%	22.6%
	[4.2; 4.4]	[13.7;14.2]	[5.9; 6.2]	[22.3; 22.9]

**Imputed linked dataset #3; *n* = 4503 of which 78 cases had an inpatient sepsis**				
	qSOFA	MEWS	SIRS	NEWS2
% of cases labeled with sepsis suspicion by respective tool [95% CI]	3.7%	12.5%	6.1%	19.4%
	[3.2; 4.3]	[11.6; 13.5]	[5.4; 6.8]	[18.3; 20.6]
Sensitivity (Se; percent, [95% CI])	23.1% [21.8; 24.3]	48.7% [47.3; 50.2]	28.2% [26.9; 29.5]	73.1% [71.8; 74.4]
Specificity (Sp; percent, [95% CI])	96.6% [96.1; 97.1]	88.1% [87.2; 89.1]	94.3% [93.6; 95.0]	81.6% [80.4; 82.7]
Area under the ROC curve (AUROC, Scores dichotomous, [CI])	0.598 [0.526; 0.670]	0.684 [0.615; 0.753]	0.613 [0.541; 0.684]	0.773 [0.716; 0.831]
Positive predictive value [95% CI]	10.7% [9.8; 11.6]	6.7% [6.0; 7.5]	8.0% [7.2; 8.8]	6.5% [5.8; 7.3]
Negative predictive value [95% CI]	98.6% [98.3; 99.0]	99.0% [98.7; 99.3]	98.7% [98.3; 99.0]	99.4% [99.2; 99.6]
Positive likelihood ratio (LR +)	6.8	4.1	5.0	4.0
Negative likelihood ratio (LR−)	0.8	0.6	0.8	0.3
	Cases labeled positive in *all* four screening tools			
% of cases labeled with sepsis suspicion by *all* four screening tools [95% CI]	0.8% [0.6; 1.1]			
Sensitivity (Se; percent, [95% CI])	7.7% [6.9; 8.5]			
Specificity (Sp; percent, [95% CI])	99.3% [99.1; 99.6]			
Area under the ROC curve (AUROC, Scores dichotomous, [CI])	0.535 [0.467; 0.603]			
Positive predictive value [95% CI]	16.7% [15.6; 17.8]			
Negative predictive value [95% CI]	98.4% [98.0; 98.8]			
	Cases labeled positive in *any* of the four screening tools			
% of cases labeled with sepsis suspicion by *any* of the four screening tools [95% CI]	24.2% [23.0; 25.5]			
Sensitivity (Se; percent, [95% CI])	76.9% [75.7; 78.2]			
Specificity (Sp; percent, [95% CI])	76.7% [75.5; 78.0]			
Area under the ROC curve (AUROC, Scores dichotomous, [CI])	0.768 [0.714; 0.823]			
Positive predictive value [95% CI]	5.5% [4.8; 6.2]			
Negative predictive value [95% CI]	99.5% [99.3; 99.7]			

*CI* confidence interval; *AUROC* area under the receiver operating characteristic curve

**Fig. 3:**
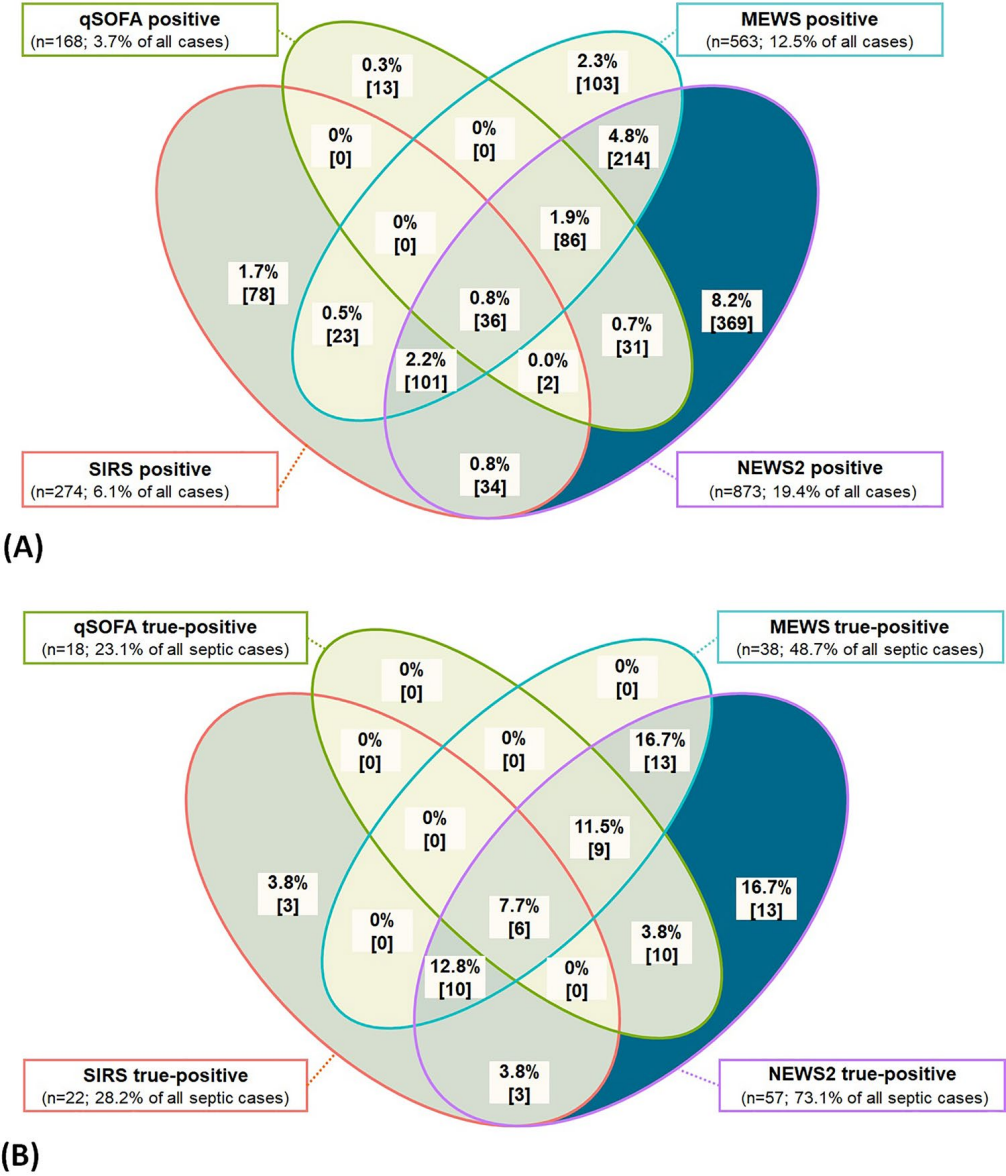
Schematic representation of intersections between screening tools (imputed, linked dataset #3; patient age ≥ 18 years). **A** Percent and in square brackets absolute number of positive screenings (consisting of true-positives and false-positives) out of *n* = 4503 cases (of those, *n* = 78 had a confirmed inpatient sepsis). **B** Percent and in square brackets absolute number of true-positive cases out of all patients with a confirmed inpatient sepsis (*n* = 78)

Screening tools differed greatly in terms of which EMS case they identified as potentially septic: Out of all EMS cases, 24.2% [23.0; 25.5%] were screening positive in *at least one* of the screening tools, but only 0.8% [0.6; 1.1] in *all* of the screening tools simultaneously (ibid.). Each tool labeled a few cases with sepsis suspicion, which no other tool did: NEWS2 was the tool with the highest percentage of *uniquely* labeling cases with sepsis suspicion (NEWS2 only: 8.2% [7.4; 9.0%]; Fig. 3A). Out of all patients with an inpatient sepsis, 16.7% [8.4; 24.9%] (*n* = 13/78) were only predicted by NEWS2 (Fig. 3B).

Key results: EMS do not sufficiently convert critical vital signs into sepsis suspicions. Screening tools help predict more septic cases, but they differ widely in terms of which case they identify as potentially septic. NEWS2 is the best tool for predicting most septic cases and with the best trade-off between true-positive and false-positive rates

## Discussion

This is the first study comparing the screening tools’ predictive ability for an inpatient sepsis based on a large, linked data set for all EMS patients. It is also the first multi-regional analysis of EMS’ sepsis incidence, case fatality, completeness of vital sign documentation and sepsis suspicion rates in Germany. The patients’ in-hospital case fatality rate and absolute number of deaths with sepsis was much higher than those for myocardial infarction and stroke, which highlights the importance of identifying sepsis by EMS providers. These results are similar to those in a U.S. study for adult, hospitalized EMS users [31].

Our observed sepsis incidence of approximately 2% was similar to that in a Canadian EMS study (2.1% [32]) and slightly lower than in other studies (with incidences of 3–4%) [11, 31, 33]. Those differences may, among others, arise from methodological disparities, as our incidence refers to *all* EMS patients (in contrast to hospitalized patients only) and different ICD coding schemes applied (e.g., ICD-9-CM-coding strategies in [31] versus ICD-10-GM in our study). In contrast to our results, in the U.S. study, sepsis incidence was slightly higher than that of myocardial infarction and stroke (ibid.).

One reason for low sepsis suspicion rates might be that health providers’ and the public’s sepsis awareness is improvable: For example, knowledge about early warning signs is lower for sepsis than for myocardial infarction and stroke [25–27, 34, 35]. At the same time, this improvable sepsis knowledge gives hope that similar quality improvement and awareness programs which have been successful for myocardial infarction and stroke (cf. [36, 37]) may also reduce the high sepsis lethality.

Studies world-wide have shown similar low documentation rates for sepsis suspicions [10, 11, 38, 39] and vital signs [23], even in samples limited to infections or sepsis [8, 24]. One study highlighted that better EMS’ documentation rates of vital signs are associated with higher sepsis suspicion rates and that many septic cases were missed when EMS did not document vital signs completely [8]. It seems plausible that incomplete vital sign assessment results in overlooking patients who are in need for screening.

At the same time, our analyses highlight that even cases with incomplete vital sign assessment should have been documented with “sepsis suspicion” more frequently (Online Resource 5, method #1): For example, applying qSOFA to the raw, unimputed data would have led to at least 2.3% of all cases screening positive. As it was not until the middle of the observation period (July 2016) that a German consensus statement clearly recommended EMS to use the qSOFA [17], this may explain some parts of the low suspicion rates. Yet, studies show that qSOFA and general sepsis symptoms are still unknown to many EMS providers world-wide [25, 27]. Just like in our study, abnormal vital signs are often not “translated” into sepsis suspicion in other countries as well [40]. It remains possible that EMS orally forwarded a sepsis impression but did not document it. Yet, low documentation rates for screening-relevant parameters, the lack of translation from alarming vital signs to a documented sepsis suspicion and surveys on EMS’ improvable knowledge about sepsis symptoms make it unlikely that orally forwarded, yet undocumented sepsis suspicions occurred frequently.

The present study highlights that the screening tools’ results differ greatly. As the qSOFA is easy to calculate and has the highest specificity and positive predictive values, it may be the best tool to quickly identify patients at high risk of having or developing sepsis. Yet, it may result in harm for septic patients, as it is the screening tool that misses the most septic cases. NEWS2 is best in recognizing as many septic patients as possible, and in keeping a good trade-off between true and false-positive rates. Its accuracy is within the range of models using artificial intelligence—though most of them are applied at later time-periods in the hospital setting, for which one could have expected increased predictive power due to more information and shorter prediction windows (cf. [41]). Given the high lethality of undetected sepsis or delayed treatment, the use of the NEWS2 may be justified despite it having the lowest specificity of all tools. Yet, in our study and in an EMS study from the U.K., NEWS2 identifies every fifth EMS patient as potentially septic [42]. This, in combination with a low positive predictive value, indicates a need for further evaluation, considering potential alarm fatigue in subsequent providers and harm for false-positive patients (cf. [43, 44]). Additionally, one ought to keep in mind that the NEWS2 is a comparably complex tool if not digitally supported [32].

In the current study and an EMS study by Lane et al. [19], MEWS or SIRS showed little practical advantage compared to qSOFA or NEWS2, as the latter had the better prediction results. MEWS’ or SIRS’ predictive ability may be better in settings where both tools can be used with the complete number of parameters.

A practical rule of thumb for EMS staff could be:

1. NEWS2 negative patients are the most *likely* to be non-septic (“rule out”),
2. For NEWS2-positive patients, sepsis should be on the priority list of differential diagnoses,
3. qSOFA-positive patients are the most *likely* to be septic, but a negative qSOFA is not suitable to dismiss a sepsis diagnosis.

The tools were tested for all adult EMS users, whereas the SSC guideline recommends sepsis screening for “acutely ill, high-risk patients” ([12], p. e1063), but does not state how to identify them. In contrast, the Royal College of Physicians’ recommendations for the NEWS2 calls for a standardized “routine recording” for *all* patients $\ge$ 16 years without pregnancy ([45], p.8). Our study with its incomplete vital sign assessments, low sepsis suspicion rates despite alarming vital signs and other studies on the rare recognition of sepsis or infection [10, 11, 32, 38] lean toward the conclusion that too many patients and valuable intervention periods could be overlooked, if EMS were asked to only screen those patients they consider acutely ill or at high risk.

Altogether, screening tools are meant to prompt additional assessments, not as a diagnostic rule. False-negative and false-positive screening results reinforce the ongoing need for expert-based decision. Nonetheless, our retrospectively calculated screening results showed that screening tools are very valuable in identifying more septic patients compared to EMS staffs’ currently documented sepsis suspicion rates.

### Strengths and limitations

One strength of our study is the comparison of all screening tools using the same methods and dataset. This strength becomes especially apparent as the study showed that different methods to handle EMS documentation deficiencies influence screening results (see Online Resource 5). At the same time, imputation is always inferior to a trustworthy, complete patient documentation by EMS. Yet, as documentation deficiencies are common in other studies as well [8, 23, 24], imputation might currently be the best method available. Especially treating unrecorded parameters as “healthy” values likely leads to an underestimation of screening tools’ predictive ability: This method resulted in the lowest sensitivities compared to all other methods for missing values. Another study also found more prominent documentation deficiencies in cases which were judged as “urgent journey” by the dispatch center [8]. There are indicators that the imputation achieved its aim and that the screening results are plausible:

1. The screening results based on the imputed results equal the tendencies found in other studies, for example high specificity for qSOFA and high sensitivity for NEWS2 [19, 46].
2. The rate of EMS cases labeled as septic by NEWS2 is very similar to an English study [42]. Since NEWS2 bases on many parameters that are also used for the three other screening tools, there is a high chance that the quality of imputation was similarly accomplished for all screening tools.
3. Own sensitivity analysis using different methods for imputed versus non-imputed vital signs revealed that the overall ranking remains stable, with qSOFA being the most specific and NEWS2 being the most sensitive tool.

Apart from a systematic literature search which yielded no eligible study (Online Resource 6), our general research in the field identified only one other study which compared all screening tools, but it was limited to patients with infections diagnosed in the Emergency Department (ED) [19]. As EMS rarely recognize infections [32], it seems beneficial that our study is the first to include all patients independent from any presumptions or preliminary diagnoses by EMS.

For the emergency physicians’ data, the former coding standard only allowed to extract how often they suspected a septic shock: Since their additional free text fields were unavailable for analysis, their suspicion rates for sepsis without shock remains unknown.

The study design has the limitation that it does not allow to extract how often sepsis was already present or fully manifested during EMS use: The screening tools were only tested for predicting an inpatient sepsis. At the same time, it is known that the majority of sepsis starts in the community setting and is present on admission [5, 47, 48]. We cannot rule out that some septic cases were missed, e.g., due to patients refusing to be conveyed to hospitals or deaths on-site resulting in not receiving an inpatient sepsis diagnosis.

As for this “gold standard”, the inpatient sepsis diagnosis, one should take into consideration that diagnosing sepsis remains a challenging task altogether (cf. [49], p. 807). Even retrospective manual chart reviews by sepsis experts do not lead to 100% interrater-agreement for inpatient sepsis diagnoses ([30, 50] for sepsis-2-definition). Due to our large dataset, manual chart review to label (non)septic cases was not feasible. Health claims data have limitations, e.g., coding strategies are heterogeneous depending on the documenting provider [29, 51, 52]. A strength of our study is using data from several health insurance companies billing multiple hospitals, dampening the variability in sepsis labeling among hospitals. Out of a variety of ICD coding strategies (cf. [53]), we favored the method by Fleischmann-Struzek et al. [30], refined by Schwarzkopf et al. [29], as it allowed the identification of cases according to the latest sepsis definition and reached the best balance between under- and overcoding compared to two different ICD strategies tested in two validation studies with German claims data [29, 30].

Overall, the results solely answer how valuable the tools are from a statistical point of view, but their usefulness in real-world settings is influenced by more factors (e.g., tools’ feasibility, Emergency Department staff’s reaction to screening results).

## Conclusion

Sepsis incidence rates compared to documented sepsis suspicion and alarming vital signs reveal an urgent need for educational measures—for paramedics and emergency physicians alike—to increase the likelihood of *complete* patients’ health status documentations and the translation of alarming vital signs into sepsis suspicion. Screening tools differ greatly and cannot be used interchangeably. Future guidelines should consider omitting recommendations for SIRS and MEWS for the *prehospital* setting. Though no tool provided ideal performance, we would currently recommend the NEWS2 due to its highest sensitivity and AUROC. However, as the NEWS2 leads to every fifth patient with sepsis suspicion, there is a need for real-world studies to determine its effect on all—septic and non-septic—patients. Clearly, tools with similar sensitivity but higher specificity would be helpful.

To raise awareness, it may be worth communicating that sepsis is more frequently deadly than stroke and myocardial infarction.

### Supplementary Information

Below is the link to the electronic supplementary material.

Supplementary file1 Online Resource 1: Sociodemographic characteristics & sepsis incidence, prevalence and case fatality (DOCX 41 KB)

Supplementary file2 Online Resource 2: Sepsis operationalization & sepsis suspicions (DOCX 46 KB)

Supplementary file3 Online Resource 3: Missing values & imputation (DOCX 48 KB)

Supplementary file4 Online Resource 4: Screening tools with their corresponding variables and cut-offs (DOCX 50 KB)

Supplementary file5 Online Resource 5: Screening results differentiated by different methods to treat missing values (DOCX 56 KB)

Supplementary file6 Online Resource 6: Search strategy to identify studies comparing all four screening tools (DOCX 94 KB)

## Data Availability

Due to German legislation on claims and EMS data, the data cannot be deposited publicly.

## Abbreviations

AUROC: Area under the receiver operating characteristic curve
CI: Confidence interval
ED: Emergency department
EMS: Emergency medical service
EP: Emergency physicians
ICD: International Statistical Classification of Diseases and Related Health Problems
LR +: Positive likelihood ratio
LR−: Negative likelihood ratio
MEWS: Modified early warning score
NEWS2: National early warning score 2
PM: Paramedics
SIRS: Systemic inflammatory response syndrome
Se: Sensitivity
Sp: Specificity
qSOFA: Quick sequential [sepsis-related] organ failure assessment
