# Supplementary material for: Sepsis incidence, suspicion, prediction and mortality in emergency medical services: a cohort study related to the current international sepsis guideline
Source: Infection. 2024 Feb 19;52(4):1325–35. doi: 10.1007/s15010-024-02181-5 (PMC11288994; doi:10.1007/s15010-024-02181-5)
Supplement: Supplementary file 1 — Supplementary file1 Online Resource 1: Sociodemographic characteristics & sepsis incidence, prevalence and case fatality (DOCX 41 KB) [file 15010_2024_2181_MOESM1_ESM.docx]

# Online Resource 1: Sociodemographic Characteristics & Sepsis Incidence, Prevalence and Case fatality

All EMS cases of dataset #1-3 may have been followed by any kind of care (e.g., care on site only/no transportation, inpatient or outpatient care).

In dataset #1, sub-samples for case fatality were limited to cases who had either an ongoing health insurance status with one insurer or died within the respective observation period past the EMS use. The 30-day case fatality also covered deaths past hospitalization. As claims data were available until December 31st 2017, individual follow-up periods for EMS cases’ mortality and inpatient diagnoses may have been based on data from 2017.

Within dataset #2, paramedics’ and emergency physicians’ documentation referred to district EMS cases: For regions for which this study acquired both paramedics’ and emergency physicians’ data, it was standard for the paramedic not to hand in any documentation if an emergency physician was present; in all other regions for which no emergency physician data could be acquired, paramedics were supposed to hand in their own documentation of the complete case independent of whether an emergency physician was present or not.

To be linkable, respectively to be part of dataset #3, the EMS cases had to cover insureds of the ten health insurance companies who used EMS in Bavaria or Baden-Württemberg. Only data filled out by paramedics was linkable with health claims data. Data from other federal states could not be used due to federal laws or lack of digitalized EMS documentation [1].

“Age” was based on the year 2016 (= year of the EMS case) minus year of birth. Age calculation for the linked dataset #3 was based on information from the health insurance data. Tab. 1 displays sociodemographic characteristics on patient-level, Tab. 2 and 3 on case-level.

**Tab.: 1 Sociodemographic characteristics of EMS patients (for dataset #2 we do not report sociodemographic as many patients lacked person-identifying pseudonym)**

|  | **Age** | | **Female** |
| --- | --- | --- | --- |
| **Dataset #1:**  Health claims data, n=173,579 patients | Five-Number Summary | mean; (LB95; UB95) | %;  (LB95; UB95) |
|  | 0;35;59;77;109 | 55.4; (55.2; 55.5) | 47.7%;  (47.5%; 48.0%) |
|  | n=173,535  (excl. missings: n=44) | | n=173,535  (excl. missings: n=44) |
| **Dataset #3:** Linked health claims data + EMS data  n=4,780 patients | 0;33;56;76;102 | 53.7; (52.9; 54.4) | 46.5%;  (45.1%; 48.0%) |
|  | n=4,780 (all valid) | | n=4,780 (all valid) |

Legend:

- Five-number summary (in order): minimum, first quartile (P25), median (P50), third quartile (P75), maximum
- LB95: lower bound of 95% confidence level; UB95: upper bound of confidence level

**Tab.: 2 Sociodemographic characteristics of EMS cases (multiple counts per patient possible)**

|  | **Age** | | **Female** | **Proportion of cases with inpatient care following EMS use** |
| --- | --- | --- | --- | --- |
|  | Five-Number Summary | mean;  (LB95; UB95) | %;  (rel_LB95; rel_UB95) | %;  (rel_LB95; rel_UB95) |
| **Dataset #1:**  Health claims data, n=221,429 cases | 0;38;62;79;109 | 57.6;  (57.5; 57.7) | 47.5%;  (47.3%; 47.7%) | 58.3%;  (58.1%; 58.5%) |
|  | n=221,368 (excl. missings: n=61) | | n=221,368 (excl. missings: n=61) | n=221,429  (all valid) |
| **Dataset #2:**  EMS data; n=110,419 cases | 0;40;66;80;116 | 59.4;  (59.3; 59.6) | 50.0%;  (49.7%; 50.3%) | Cannot be calculated |
|  | n=110,263  (excl. missings: n=156) | | n=96,413  (excl. missings: n=14.006) |  |
| **Dataset #3:** Linked health claims data + EMS data  n=5,465 cases | 0;35;58;77;102 | 55.0;  (54.4; 55.7) | 46.5%;  (45.2%; 47.8%) | 64.4%;  (63.2%; 65.7%) |
|  | n=5,465  (all valid) | | n=5,465  (all valid) | n=5,465  (all valid) |

Legend:

- Five-number summary (in order): minimum, first quartile (P25), median (P50), third quartile (P75), maximum
- LB95: lower bound of 95% confidence level; UB95: upper bound of confidence level

**Tab. 3: Sociodemographic characteristics for EMS cases with inpatient sepsis compared to cases without inpatient sepsis (multiple counts per patient possible)**

|  | **Age** | | **Female** | **Age** | | **Female** |
| --- | --- | --- | --- | --- | --- | --- |
|  | Five-Number Summary | mean;  (LB95; UB95) | %;  (LB95; UB95) | Five-Number Summary | mean;  (LB95; UB95) | %;  (LB95; UB95) |
| **Dataset #1:** Health claims data, n=221,429 cases | Cases with inpatient sepsis (n=3,470; all valid) | | | Cases without inpatient sepsis (n=217,898, excl. missings: n=61) | | |
|  | 0; 64.0; 75.0; 82.0; 98.0 | 71.8 (71.3; 72.3) | 37.9% (36.3; 39.5) | 0;38.0;62.0;79.0;109.0 | 57.4;  (57.3; 57.5) | 47.6% (47.4; 47.8) |
| **Dataset #3:** Linked health claims data + EMS data  n=5,465 cases | Cases with inpatient sepsis (n=87; all valid) | | | Cases without inpatient sepsis (n=5,378, all valid) | | |
|  | 26.0; 62.0; 73.0; 79.0; 94.0 | 70.5 (67.5; 73.4) | 32.2% (23.1; 42.5) | 0; 34.0; 57.0; 77.0; 102 | 54.8  (54.1; 55.4) | 46.7% (45.4; 48.1) |

Legend:

- Five-number summary (in order): minimum, first quartile (P25), median (P50), third quartile (P75), maximum
- LB95: lower bound of 95% confidence level; UB95: upper bound of confidence level

**Tab. 4: Sepsis incidence (case-level; multiple counts per patient possible) and 1-year prevalence (patient-level)**

|  | **Incidence in % (CI)** |  | **1-year prevalence in % (CI)** |
| --- | --- | --- | --- |
| **Dataset #1:**  Health claims data,  n=221,429 cases | 1.6%  (1.5%; 1.6%) | **Dataset #1:** Health claims data,  n=173,579 patients | 1.9%  (1.9%; 2.0%) |
| **Dataset #3:** Linked health claims data + EMS data  n=5,465 cases | 1.6%  (1.3%; 1.9%) | **Dataset #3:** Linked health claims data + EMS data  n=4,780 patients | 1.8%  (1.5%; 2.2%) |

Legend:

CI: 95% Confidence interval

**Tab. 5: Case fatality comparison between sepsis, myocardial infarction and stroke following EMS use (Dataset #1)**

|  | **Hospital case fatality in % (CI)** | **30-day-case fatality in %**  **(CI)** |
| --- | --- | --- |
| Sepsis (n=3,465 cases) | 31.6%  (30.1; 33.2%) | 31.7%  (30.2; 33.3%) |
| Myocardial infarction (n=5,713 cases) | 11.4%  (10.6; 12.2%) | 13.4%  (12.5; 14.3%) |
| Stroke (n=5,891 cases) | 8.7%  (8.0; 9.5%) | 11.8%  (11.0; 12.7%) |

Legend:

CI: 95% Confidence interval

References

1. Piedmont S, Brammen D, Branse D, Focke K, Kast W, Robra B-P. Auf dem Weg zur integrierten Qualitätssicherung im Rettungsdienst. Notfall Rettungsmed. 2018;21:682–9. doi:10.1007/s10049-018-0440-9.
