# Supplementary material for: Sepsis incidence, suspicion, prediction and mortality in emergency medical services: a cohort study related to the current international sepsis guideline
Source: Infection. 2024 Feb 19;52(4):1325–35. doi: 10.1007/s15010-024-02181-5 (PMC11288994; doi:10.1007/s15010-024-02181-5)
Supplement: Supplementary file 2 — Supplementary file2 Online Resource 2: Sepsis operationalization & sepsis suspicions (DOCX 46 KB) [file 15010_2024_2181_MOESM2_ESM.docx]

Online Resource 2: ICD-based sepsis operationalization in claims data and sepsis suspicions in EMS data

# ICD based sepsis operationalization in health claims data

An EMS case is considered to result in an inpatient sepsis diagnosis, if the diagnosis belongs to a hospital stay starting on the day of EMS use.

The operationalization for ICD-10-GM codes by Fleischmann-Struzek et al. [1] as refined by Schwarzkopf et al. [2] was based on the Martin method [3]. It includes

1. explicit codes for severe sepsis and septic shock – which automatically maintain an organ dysfunction (ICD Code “R65.1”: “Systemic Inflammatory Response Syndrome of infectious origin with organ failure, severe sepsis” or “R57.2”: “septic shock”),
2. but also explicit sepsis codes (e.g., B37.7: “Candidal sepsis”) in combination with organ dysfunction codes (e.g., J960: “Acute respiratory failure”).

To be labeled as an inpatient sepsis case, it must either be based on the operationalization #1 (R65.1 or R57.2 is present) or operationalization #2 (explicit sepsis code *and* additionally a code for an organ dysfunction). In Table 1, ICD Codes “R65.1” (“Systemic Inflammatory Response Syndrome of infectious origin with organ failure, severe sepsis”) and ICD Codes “R57.2” (“septic shock”) are listed in both columns, as their sole documentation is sufficient to be considered an inpatient sepsis. The list includes codes for neonatal sepsis. Listed diagnoses can be a primary or secondary diagnosis and are extracted from health claims data.

**Table 1: ICD operationalization for sepsis**

| **Any code for explicit sepsis** | **AND** | **Any code for organ dysfunction** |
| --- | --- | --- |
| A021 |  | I959 |
| A200 |  | R578 |
| A207 |  | R579 |
| A217 |  | J960 |
| A227 |  | J969 |
| A241 |  | J80 |
| A267 |  | J984 |
| A282 |  | R060 |
| A327 |  | R068 |
| A391 |  | F05 |
| A392 |  | G931 |
| A393 |  | G934 |
| A394 |  | R40 |
| A40 |  | N17 |
| A41 |  | N19 |
| A427 |  | E872 |
| A483 |  | D65 |
| A499 |  | D688 |
| A548 |  | D689 |
| B007 |  | D695 |
| B376 |  | D696 |
| B377 |  | K720 |
| B49 |  | K727 |
| P36 |  | K762 |
| R650 |  | K763 |
| R651 |  | R651 |
| R572 |  | R572 |

# Operationalization of sepsis suspicions during EMS care

EMS from the federal state Baden-Württemberg stated to have used the version MIND3BW which would imply that the code 1803 refers to “septic shock” only. With this MIND3BW standard, there was no other option to check “sepsis” or “severe sepsis” (we refrain from citations as the MIND3BW is not publicly available on any website anymore). For one EMS region in Baden-Württemberg, free text fields on preliminary diagnoses by paramedics were provided in addition to the standardized codes.

For the paramedics’ data from Bavaria, data owner stated to have used the MIND3.1, which implies that the code 1803 refers to “high fever infection/sepsis/septic shock” [4].

References

1. Fleischmann-Struzek C, Thomas-Rüddel DO, Schettler A, Schwarzkopf D, Stacke A, Seymour CW, Haas C, Dennler U, Reinhart K (2018) Comparing the validity of different ICD coding abstraction strategies for sepsis case identification in German claims data. PloS one 13(7):e0198847

2. Schwarzkopf D, Rose N, Fleischmann-Struzek C, Boden B, Dorow H, Edel A, Friedrich M, Gonnert FA, Götz J, Gründling M, Heim M, Holbeck K, Jaschinski U, Koch C, Künzer C, Le Ngoc K, Lindau S, Mehlmann NB, Meschede J, Meybohm P, Ouart D, Putensen C, Sander M, Schewe J-C, Schlattmann P, Schmidt G, Schneider G, Spies C, Steinsberger F, Zacharowski K, Zinn S, Reinhart K (2023) Understanding the biases to sepsis surveillance and quality assurance caused by inaccurate coding in administrative health data. Infection

3. Martin GS, Mannino DM, Eaton S, Moss M (2003) The epidemiology of sepsis in the United States from 1979 through 2000. The New England journal of medicine 348(16):1546–1554

4. o.A. MINIMALER NOTFALLDATENSATZ MIND3.1 Codierte Werte. https://www.divi.de/joomlatools-files/docman-files/mind-notfalleinsatzprotokoll/MIND3.1_Codes20170227.pdf. Accessed 25 September 2023
