# Supplementary material for: Sepsis incidence, suspicion, prediction and mortality in emergency medical services: a cohort study related to the current international sepsis guideline
Source: Infection. 2024 Feb 19;52(4):1325–35. doi: 10.1007/s15010-024-02181-5 (PMC11288994; doi:10.1007/s15010-024-02181-5)
Supplement: Supplementary file 3 — Supplementary file3 Online Resource 3: Missing values & imputation (DOCX 48 KB) [file 15010_2024_2181_MOESM3_ESM.docx]

# Online Resource 3: Missing Values and Imputation

In this section, we report the imputation method and results for dataset #2 (EMS data). The imputation method is the same for dataset #3 which relies on those EMS cases that were linkable to health claims data.

# Missing values

To assess the completeness of sepsis screening-relevant parameters (e.g., body temperature) for EMS patients of all ages, we transformed the information “not measured”/”not measurable” to missing values. Medically implausible data were transformed to missing values. This was only necessary for a temperature outside of 30-43° Celsius (which applied to 0,09% of all cases) and respiratory rates apart from 0-150 per minute to missing values (implausible: 0,01% of all cases).

# Imputation

Imputation and the analysis of screening results was limited to adult patients as younger patients tend to have other thresholds for critical vital signs and the screening tools to be tested are recommended for adults. Missing value analysis revealed no monotone pattern. Missing values were imputed using multiple imputation for all screening relevant parameters (e.g., temperature, heart rate) listed in Table 1. As not all data sources allowed to distinguish completeness rates for requiring supplemental oxygen, this parameter’s rate of missing values remains unreported.

If parameters were measured up to two times per EMS case and screening tool relied on minimum or maximum of those variables, we imputed both minimum and maximum of those parameters per EMS.

As all variables contained some missing values, all variables were used as independent and dependent variables, respectively, sorted by decreasing completeness rates for documentation and undergoing five imputations. Continuous variables were imputed on the raw scale independent from their (sometimes skewed) distribution. Glasgow Coma scale was used as metric variable. All metric variables were imputed with the constraints to be rounded to the same decimal place as the original data. Consciousness (including values such as “alert”) was used as categorial variable. Conducting multiple imputation with the software SPSS, metric variables were imputed using linear regressions, the categorial variable using logistic regression.

If not even one of the variables listed in Table 1 was present per EMS case or patients were younger than 18, no imputation occurred. Consequently, 91,884 cases (out of all original 110,419 cases in dataset #2) had original or imputed values for screening relevant variables after imputation.

In dataset #3, there were 9 inpatient sepsis cases without any EMS documentation of screening-relevant parameters.

# Calculation of screening results based on imputation

Screening results were based on the variables’ rounded averages of all five imputations. As the data set did not allow identification whether the NEWS-2 relevant variable “supplemental oxygen” was missing, we refrained from its imputation and added two points to the NEWS-2 score in case “supplemental oxygen” was already documented in the original data set.

**Table 1: Descriptive statistics for screening relevant variables comparing observed versus imputed data
(belonging to n=91,884 EMS cases of ≥ 18 years; Dataset #2)**

|  | **Observed** (due to missing values n varies per variable) | | | | | **Imputed** (n valid=91,884 for each variable) | | | |
| --- | --- | --- | --- | --- | --- | --- | --- | --- | --- |
|  | n valid | mean | SD | Min | Max | mean | SD | Min | Max |
| Temperature maximum | 18,065 | 36.95 | 1.21 | 30.00 | 42.00 | 36.94 | 0.75 | 30.0 | 42.0 |
| Temperature minimum | 18,065 | 36.94 | 1.22 | 30.00 | 42.00 | 36.93 | 0.70 | 30.0 | 42.0 |
| Respiratory rate maximum | 28,844 | 14.69 | 5.60 | 0.00 | 150.00 | 14.74 | 3.87 | 0 | 150 |
| Respiratory rate minimum | 28,844 | 14.02 | 4.67 | 0.00 | 150.00 | 14.05 | 3.25 | 0 | 150 |
| Oxygen saturation | 82,339 | 94.25 | 8.92 | 0.00 | 100.00 | 94.15 | 8.72 | 0 | 108.4 |
| Systolic blood pressure maximum | 83,446 | 145.90 | 31.84 | 0.00 | 300.00 | 145.67 | 30.78 | 0 | 300 |
| Systolic blood pressure minimum | 83,446 | 136.97 | 30.08 | 0.00 | 300.00 | 136.70 | 29.19 | 0 | 300 |
| Heart rate maximum | 84,935 | 89.48 | 24.23 | 0.00 | 300.00 | 89.43 | 23.50 | 0 | 300 |
| Heart rate minimum | 84,935 | 83.53 | 21.39 | 0.00 | 299.00 | 83.44 | 20.79 | 0 | 299 |
| GCS minimum | 79,894 | 14.29 | 2.28 | 3.00 | 15.00 | 14.23 | 2.27 | 2.8 | 17.6 |
|  | N valid | % | CI  Lb | CI Ub |  | % | CI  Lb | CI  Ub |  |
| Consciousness | 88,651 | 86.6% alert | 86.3% | 86.8% | - | 85.9% alert | 85.7% | 86.1% | - |
|  |  | 6.9% voice | 6.7% | 7.1% |  | 7.5% voice | 7.3% | 7.6% |  |
|  |  | 1.9% pain | 1.8% | 2.0% |  | 2.0% pain | 1.9% | 2.1% |  |
|  |  | 4.7% unresponsive | 4.5% | 4.8% |  | 4.6% unresponsive | 4.5% | 4.8% |  |

Legend:

SD: Standard derivation; Min: Minimum; Max: Maximum; CI: Confidence interval; Lb: lower bound; Ub: upper bound; GCS: Glasgow Coma Scale

Methodological sidenotes on the imputation:

The methodology for imputations (and the evaluation of imputation quality in particular) is steadily evolving and offering a variety of options to analyze and visualize imputation quality (cf. [1]). For example, Stuart et al. propose *“flagging variables if the ratio of variances of the observed and imputed values is less than 0.5 or greater than 2, or if the absolute difference in means is greater than two standard deviations.”* ([2], cited by [1], p.7). As can be seen in the table above, we observe little variance between original and imputed data.

Our imputed maximums for oxygen saturation and GCS are outside of physiologically plausible ranges. Nguyen et al. ([1], p.4) conclude based on [3, 4]*: “Simulation studies have indicated that it is not essential that imputed values fall within plausible or possible ranges (…)”.* Thus, we did not preset boundaries on minimum and maximum values. When calculating screening results, patients with an imputed oxygen saturation above 100% or GCS above 15 were considered to have normal, healthy states.

References

1. Nguyen CD, Carlin JB, Lee KJ (2017) Model checking in multiple imputation: an overview and case study. Emerging themes in epidemiology 14:8

2. Stuart EA, Azur M, Frangakis C, Leaf P (2009) Multiple imputation with large data sets: a case study of the Children's Mental Health Initiative. American journal of epidemiology 169(9):1133–1139

3. Hippel PT von (2013) Should a Normal Imputation Model be Modified to Impute Skewed Variables? Sociological Methods & Research 42(1):105–138

4. Rodwell L, Lee KJ, Romaniuk H, Carlin JB (2014) Comparison of methods for imputing limited-range variables: a simulation study. BMC medical research methodology 14:57
