# Supplementary material for: Sepsis incidence, suspicion, prediction and mortality in emergency medical services: a cohort study related to the current international sepsis guideline
Source: Infection. 2024 Feb 19;52(4):1325–35. doi: 10.1007/s15010-024-02181-5 (PMC11288994; doi:10.1007/s15010-024-02181-5)
Supplement: Supplementary file 4 — Supplementary file4 Online Resource 4: Screening tools with their corresponding variables and cut-offs (DOCX 50 KB) [file 15010_2024_2181_MOESM4_ESM.docx]

# Online Resource 4: Screening tools with their corresponding variables and cut-offs

For cases with two measurements of the same parameter (e.g., respiration rates measured upon EMS arrival and upon transferal to the hospital), we used the worst/most unhealthy state and corresponding score weight. For example, using the NEWS2 score, if a patient had a tachypnea of 22 breaths per minute in the first assessment, this case severity equals 2 points. If the same EMS case also had a bradypnea of 11 breaths per minute during the second assessment, this severity equals 1 point. In consequence, the NEWS2 calculation for the respiration rate bases on the highest (“unhealthy”) point score during the entire EMS cases which, in this example, equals 2 points for the parameter respiration rates.

Each other parameter (e.g., blood pressure) is calculated in the same manner. All parameters’ points are added together to the screening tools’ sum score.

# qSOFA

qSOFA positive with two or more of:

| **Seymour et al. [1]** | **Prehospitally available data  (version used in present paper)** |
| --- | --- |
| - systolic hypotension [≤100 mm Hg] | Used as in original version |
| - tachypnea [≥22/min] |  |
| - altered mentation |  |

# SIRS criteria

SIRS-positive with two or more of:

| **Bone at al. [2]** | **Prehospitally available data  (version used in present paper)** |
| --- | --- |
| - Temperature >38°C or <36°C | Used as in original version |
| - Heart rate >90/min |  |
| - Respiratory rate >20/min or PaCO_2_ <32 mm Hg (4.3 kPa) | only used respiratory rate as PaCo_2_ was not available |
| - White blood cell count >12 000/mm3 or <4000/mm³ or 10% immature bands | –  not used/not available in EMS data |

# Modified Early Warning Score (MEWS)

MEWS-positive with a sum score of 4 points or more:

| **Gardner-Thorpe et al. [3]** | | | | | | | | **Prehospitally available data  (version used in present paper)** |
| --- | --- | --- | --- | --- | --- | --- | --- | --- |
| **Score** | **3** | **2** | **1** | **0** | **1** | **2** | **3** |  |
| Respiratory rate (min) |  | ≤ 8 |  | 9–14 | 15–20 | 21–29 | > 29 | Used as in original version |
| Heart rate (min) |  | ≤ 40 | 41–50 | 51–100 | 101–110 | 111–129 | > 129 |  |
| Systolic BP (mmHg) | ≤ 70 | 71–80 | 81–100 | 101–199 |  | ≥ 200 |  |  |
| Temperature (°C) |  | ≤ 35 | 35.1–36.0 | 36.1–38 | 38.1–38.5 | ≥ 38.6 |  |  |
| Neurological |  |  |  | Alert | Reacting to voice | Reacting to pain | Unresponsive |  |
| Urine Output |  |  |  |  |  |  |  | –  not used/not available in EMS data |

# NEWS2

NEWS2-positive with a sum score of 5 points or more [4] as recommended by the NHS England Sepsis guidance implementation advice for adults [5];

The National Health Service’s guidelines on NEWS further recommends: *“Clinicians assessing patients with a NEWS score of less than 5 should still be aware of the risk of sepsis and should specifically look for: a single NEWS parameter of 3 (…)”* ([5], p.8). In the current study, including those patients with a score of 3 for a *single* parameter as well would have led to an even higher percentage of cases labeled as septic positive (25.0%) and a lower AUROC (50.0% [CI: 42.2; 57.8%]) compared to only labeling septic patients with an aggregated NEWS2 score of ≥5. Thus, we refrained from labeling cases as “sepsis positive” due to an extreme value of 3 in a *single* parameter.

Parameters were used as in original version by Royal College of Physicians, SpO_2_ relied on scale 1.

| **Royal College of Physicians [4]** | | | **Prehospitally available data  (version used in present paper)** |
| --- | --- | --- | --- |
|  | **Value** | **Points** |  |
| Respirations (breaths/min) | ≥25 | 3 | Used as in original version, only for the parameter “consciousness” the value “confusion” was not part of the documentation standards |
|  | 21-24 | 2 |  |
|  | 12-20 | 0 |  |
|  | 9-11 | 1 |  |
|  | ≤8 | 3 |  |
| SpO_2_ Scale1  (oxygen saturation in %) | ≥96 | 0 |  |
|  | 94-95 | 1 |  |
|  | 92-93 | 2 |  |
|  | ≤91 | 3 |  |
| Air or oxygen | O_2_ L/min | 2 |  |
| Blood pressure | ≥ 220 | 3 |  |
|  | 111-219 | 0 |  |
|  | 101-110 | 1 |  |
|  | 91-100 | 2 |  |
|  | ≤90 | 3 |  |
| Pulse (beats/min) | ≥131 | 3 |  |
|  | 121-130 | 2 |  |
|  | 111-120 | 1 |  |
|  | 51-110 | 0 |  |
|  | 41-50 | 1 |  |
|  | ≤40 | 3 |  |
| Consciousness | Alert | 0 |  |
|  | Confusion | 3 |  |
|  | Verbal |  |  |
|  | Pain |  |  |
|  | Unresponsive |  |  |
| Temperature (°C) | ≥ 39.1 | 2 |  |
|  | 38.1-39.0 | 1 |  |
|  | 36.1-38.0 | 0 |  |
|  | 35.1-36.0 | 1 |  |
|  | ≤ 35.0 | 2 |  |

References

1. Seymour CW, Liu VX, Iwashyna TJ, Brunkhorst FM, Rea TD, Scherag A, Rubenfeld G, Kahn JM, Shankar-Hari M, Singer M, Deutschman CS, Escobar GJ, Angus DC (2016) Assessment of Clinical Criteria for Sepsis: For the Third International Consensus Definitions for Sepsis and Septic Shock (Sepsis-3). JAMA 315(8):762–774

2. Bone RC, Balk RA, Cerra FB, Dellinger RP, Fein AM, Knaus WA, Schein RM, Sibbald WJ (1992) Definitions for sepsis and organ failure and guidelines for the use of innovative therapies in sepsis. The ACCP/SCCM Consensus Conference Committee. American College of Chest Physicians/Society of Critical Care Medicine. Chest 101(6):1644–1655

3. Gardner-Thorpe J, Love N, Wrightson J, Walsh S, Keeling N (2006) The value of Modified Early Warning Score (MEWS) in surgical in-patients: a prospective observational study. Annals of the Royal College of Surgeons of England 88(6):571–575

4. Royal College of Physicians NEWS2: Chart 3_NEWS observation chart. https://www.rcplondon.ac.uk/file/9436/download. Accessed 7 December 2021

5. NHS England Sepsis guidance implementation advice for adults. https://www.england.nhs.uk/wp-content/uploads/2017/09/sepsis-guidance-implementation-advice-for-adults.pdf. Accessed 9 September 2022
