# Supplementary material for: Sepsis incidence, suspicion, prediction and mortality in emergency medical services: a cohort study related to the current international sepsis guideline
Source: Infection. 2024 Feb 19;52(4):1325–35. doi: 10.1007/s15010-024-02181-5 (PMC11288994; doi:10.1007/s15010-024-02181-5)
Supplement: Supplementary file 5 — Supplementary file5 Online Resource 5: Screening results differentiated by different methods to treat missing values (DOCX 56 KB) [file 15010_2024_2181_MOESM5_ESM.docx]

# Online Resource 5: Screening results differentiated by different methods to treat missing values

# Method

Studies on the predictive ability of screening tools use a variety of methods to handle EMS’ documentation deficiencies. To allow comparability between studies, we report screening results using different methods of handling missing values for screening relevant variables such as temperature or heart rate:

- Method #1: All missing values were considered as normal/healthy values
- Method #2: Eligible cases had to have at least two screening-relevant variables documented in *all* screening tools alike
- Method #3: Eligible cases had to have at least two variables filled which are necessary for the *respective* screening tool
- Method #4: Missing values were imputed using multiple imputation (see Appendix 3) and main paper

All methods enabled EMS cases to reach a score threshold that either equals positive or negative screening results.

The sepsis guidelines mention qSOFA, MEWS, SIRS and NEWS2 for adults [1], whereas the pediatric guidelines do not recommend any specific tool [2]. Thus, the following results base on persons age ≥18 only.

# Results

The following results base on the linked EMS + health claims data set (dataset #3) that allowed analyses of the screening tools’ ability to predict an inpatient sepsis. Independent of how missing values were treated, qSOFA had the highest specificity and positive predictive value, while NEWS2 had the highest sensitivity, negative predictive value and area under the ROC curve (AUROC) of all tools (see Tab. 1).

**Tab. 1: Screening results with qSOFA, MEWS, SIRS and NEWS2 differentiated by method for treating missing values (varying sample sizes, based on linked dataset #3; patient age ≥ 18 years)**

|  | **Method #1** | | | | **Method #2** | | | | **Method #3** | | | | **Method #4** | | | |
| --- | --- | --- | --- | --- | --- | --- | --- | --- | --- | --- | --- | --- | --- | --- | --- | --- |
|  | Cases with missing values treated as “healthy” values (n=4,979) | | | | Cases with at least two screening-relevant variables per tool filled in *all* screening tools (n=2,061) | | | | Cases with at least two screening-relevant variables filled for the *respective* screening tool | | | | Cases with imputed data (n=4,503) | | | |
|  | **qSOFA** | **MEWS** | **SIRS** | **NEWS2** | **qSOFA** | **MEWS** | **SIRS** | **NEWS2** | **qSOFA** | **MEWS** | **SIRS** | **NEWS2** | **qSOFA** | **MEWS** | **SIRS** | **NEWS2** |
|  |  |  |  |  |  |  |  |  | (n= 3,873) | (n= 4,235) | (n= 2,101) | (n= 4,258) |  |  |  |  |
| % of positive screening results per respective tool | 2.3% | 6.6% | 3.7% | 15.5% | 4.0% | 11.6% | 8.6% | 22.0% | 3.0% | 7.8% | 8.7% | 18.1% | 3.7% | 12.5% | 6.1% | 19.4% |
|  | [1.9; 2.7] | [5.9; 7.3] | [3.1; 4.2] | [14.5; 16.5] | [3.1; 4.8] | [10.2; 13.0] | [7.4; 9.8] | [20.2; 23.8] | [2.4; 3.5] | [7.0; 8.6] | [7.5; 9.9] | [17.0; 19.3] | [3.2; 4.3] | [11.6; 13.5] | [5.4; 6.8] | [18.3; 20.6] |
| Sensitivity | 18.4% | 37.9% | 21.8% | 63.2% | 26.0% | 56.0% | 38.0% | 84.0% | 23.2% | 44.0% | 36.5% | 73.3% | 23.1% | 48.7% | 28.2% | 73.1% |
| (Se; %) | [17.3; 19.5] | [36.6; 39.3] | [20.7; 23.0] | [61.9; 64.6] | [24.1; 27.9] | [53.9; 58.1] | [35.9; 40.1] | [82.4; 85.6] | [21.9; 24.5] | [42.5; 45.5] | [34.5; 38.6] | [72.0; 74.7] | [21.8; 24.3] | [47.3; 50.2] | [26.9; 29.5] | [71.8; 74.4] |
| Specificity | 98.0% | 93.9% | 96.7% | 85.4% | 96.6% | 89.5% | 92.1% | 79.6% | 97.4% | 92.9% | 92.0% | 82.9% | 96.6% | 88.1% | 94.3% | 81.6% |
| (Sp; %) | [97.6; 98.4] | [93.3; 94.6] | [96.2; 97.2] | [84.4; 86.3] | [95.8; 97.4] | [88.2; 90.8] | [90.9; 93.3] | [77.8; 81.3] | [96.9; 97.9] | [92.1; 93.7] | [90.9; 93.2] | [81.8; 84.0] | [96.1; 97.1] | [87.2; 89.1] | [93.6; 95.0] | [80.4; 82.7] |
| Positive predictive value | 13.9% | 10.0% | 10.4% | 7.1% | 15.9% | 11.7% | 10.7% | 9.3% | 13.9% | 10.0% | 10.4% | 7.1% | 10.7% | 6.7% | 8.0% | 6.5% |
| Negative predictive value | 98.5% | 98.8% | 98.6% | 99.2% | 98.1% | 98.8% | 98.4% | 99.5% | 98.6% | 98.9% | 98.3% | 99.4% | 98.6% | 99.0% | 98.7% | 99.4% |
| Area under the ROC curve | 0.582 | 0.659 | 0.593 | 0.743 | 0.613 | 0.728 | 0.650 | 0.818 | 0.603 | 0.684 | 0.643 | 0.781 | 0.598 | 0.684 | 0.613 | 0.773 |
| (AUROC, Scores dichotomous) | [0.514; 0.650] | [0.591; 0.728] | [0.525; 0.660] | [0.683; 0.803] | [0.522; 0.704] | [0.643; 0.812] | [0.561; 0.740] | [0.758; 0.877] | [0.526; 0.680] | [0.612; 0.757] | [0.555; 0.731] | [0.723; 0.840] | [0.526; 0.670] | [0.615; 0.753] | [0.541; 0.684] | [0.716; 0.831] |
| Positive Likelihood Ratio (LR+) | 9.1 | 6.3 | 6.6 | 4.3 | 7.6 | 5.3 | 4.8 | 4.1 | 8.9 | 6.2 | 4.6 | 4.3 | 6.8 | 4.1 | 5.0 | 4.0 |
| Negative Likelihood Ratio (LR-) | 0.8 | 0.7 | 0.8 | 0.4 | 0.8 | 0.5 | 0.7 | 0.2 | 0.8 | 0.6 | 0.7 | 0.3 | 0.8 | 0.6 | 0.8 | 0.3 |
| % of positive cases in *all* four screenings tools | 0.5% | | | | 1.2% | | | | Not applicable due to different sample sizes per tool | | | | 0.8% | | | |
|  | (Se: 5.7%; Sp: 99.6%; PPV: 20.8%; NPV: 98.3%, AUROC: 0.527) | | | | (Se: 10.0%; Sp: 99.1%; PPV: 20.8%; NPV: 97.8%; AUROC: 0.545) | | | |  |  |  |  | (Se: 7.7%; Sp: 99.3%; PPV: 16.7%; NPV: 98.4%, AUROC: 0.535) | | | |
| % of positive cases in *any* of the four screenings tools | 17.4% | | | | 26.2% | | | | Not applicable due to different sample sizes per tool | | | | 24.2% | | | |
|  | (Se: 65.5%; Sp: 83.5%; PPV: 6.6%; NPV: 99.3%, AUROC: 0.745) | | | | [Se: 88.0%; Sp: 75.4%, PPV: 8.2%; NPV: 99.6%; AUROC: 0.817) | | | |  |  |  |  | (Se: 76.9%; Sp: 76.7%; PPV: 5.5%; NPV: 99.5%, AUROC: 0.768) | | | |

References

1. Evans L, Rhodes A, Alhazzani W, Antonelli M, Coopersmith CM, French C, Machado FR, Mcintyre L, Ostermann M, Prescott HC, Schorr C, Simpson S, Wiersinga WJ, Alshamsi F, Angus DC, Arabi Y, Azevedo L, Beale R, Beilman G, Belley-Cote E, Burry L, Cecconi M, Centofanti J, Coz Yataco A, Waele J de, Dellinger RP, Doi K, Du B, Estenssoro E, Ferrer R, Gomersall C, Hodgson C, Hylander Møller M, Iwashyna T, Jacob S, Kleinpell R, Klompas M, Koh Y, Kumar A, Kwizera A, Lobo S, Masur H, McGloughlin S, Mehta S, Mehta Y, Mer M, Nunnally M, Oczkowski S, Osborn T, Papathanassoglou E, Perner A, Puskarich M, Roberts J, Schweickert W, Seckel M, Sevransky J, Sprung CL, Welte T, Zimmerman J, Levy M (2021) Surviving Sepsis Campaign: International Guidelines for Management of Sepsis and Septic Shock 2021. Critical care medicine 49(11):e1063-e1143

2. Weiss SL, Peters MJ, Alhazzani W, Agus MSD, Flori HR, Inwald DP, Nadel S, Schlapbach LJ, Tasker RC, Argent AC, Brierley J, Carcillo J, Carrol ED, Carroll CL, Cheifetz IM, Choong K, Cies JJ, Cruz AT, Luca D de, Deep A, Faust SN, Oliveira CF de, Hall MW, Ishimine P, Javouhey E, Joosten KFM, Joshi P, Karam O, Kneyber MCJ, Lemson J, MacLaren G, Mehta NM, Møller MH, Newth CJL, Nguyen TC, Nishisaki A, Nunnally ME, Parker MM, Paul RM, Randolph AG, Ranjit S, Romer LH, Scott HF, Tume LN, Verger JT, Williams EA, Wolf J, Wong HR, Zimmerman JJ, Kissoon N, Tissieres P (2020) Surviving Sepsis Campaign International Guidelines for the Management of Septic Shock and Sepsis-Associated Organ Dysfunction in Children. Pediatric critical care medicine: a journal of the Society of Critical Care Medicine and the World Federation of Pediatric Intensive and Critical Care Societies 21(2):e52-e106
