# Supplementary material for: Sepsis incidence, suspicion, prediction and mortality in emergency medical services: a cohort study related to the current international sepsis guideline
Source: Infection. 2024 Feb 19;52(4):1325–35. doi: 10.1007/s15010-024-02181-5 (PMC11288994; doi:10.1007/s15010-024-02181-5)
Supplement: Supplementary file 6 — Supplementary file6 Online Resource 6: Search strategy to identify studies comparing all four screening tools (DOCX 94 KB) [file 15010_2024_2181_MOESM6_ESM.docx]

# Online Resource 6: Search strategy to identify studies comparing all four screening tools

# Method

We systematically screened the literature whether there was any published study that covered all four screening tools simultaneously and in the setting of Emergency Medical Services or, more broadly, prehospital care. The basic concept to identify studies is depicted in Figure 1.


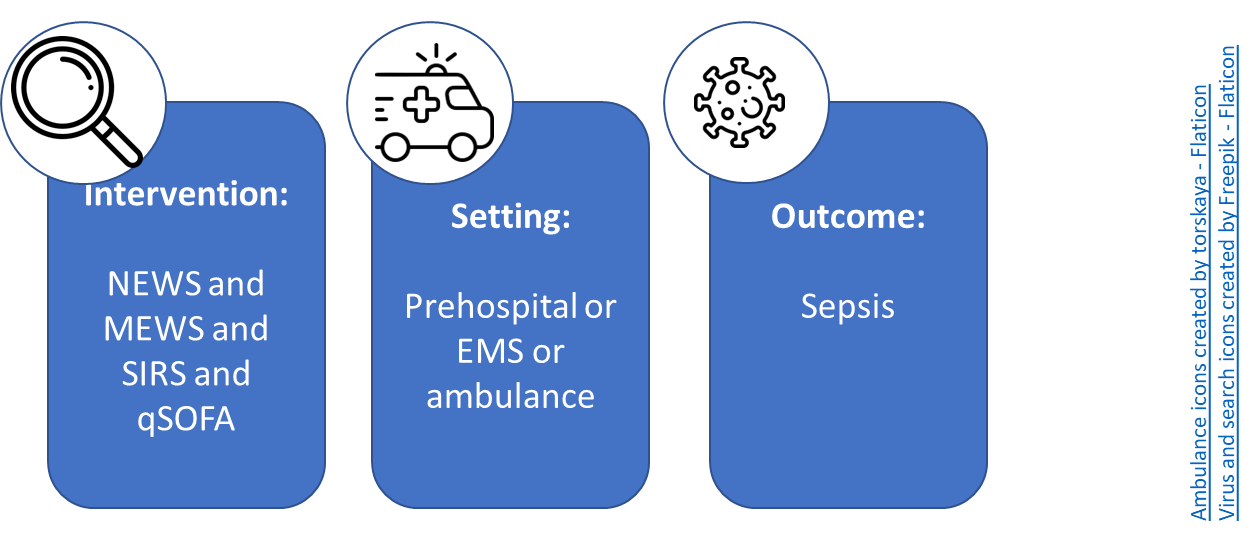


**Figure 1: General search concept**

## PUBMED: Search #1

**Component 1: INTERVENTION**

(NEWS [Text Word]) OR (NEWS-2 [Text Word]) OR (National early warning score* [Text Word]) AND

(MEWS [Text Word]) OR (Modified Early Warning Score* [Text Word]) AND

(qSOFA [Text Word]) OR (quick Sequential Organ Failure Assessment* [Text Word]) OR (quick Sepsis-related Organ Failure Assessment* [Text Word]) OR (quick Sequential Sepsis-related Organ Failure Assessment* [Text Word]) AND

(Systemic Inflammatory Response Syndrome [Text Word]) OR (SIRS [Text Word])

**Component 2: SETTING**

(Prehospital*[Text Word]) OR (Out of hospital [Text Word]) OR (out-of-hospital [Text Word]) OR (Emergency Medical Service*[Text Word]) OR (Medical Emergency Service* [Text Word]) OR (EMS [Text Word]) OR (Ambulances[MeSH Terms]) OR (ambulance*[Text Word]) OR (paramedic*[Text Word]) OR (Emergency medical technician[MeSH Terms]) OR (emergency medical technician*[Text Word])

**Component 3: Outcome**

(sepsis[Text Word]) OR (septic*[Text Word])

**🡪 Version for quick entry:**

((NEWS [Text Word]) OR (NEWS-2 [Text Word]) OR (National early warning score* [Text Word]) AND (MEWS [Text Word]) OR (Modified Early Warning Score* [Text Word]) AND (qSOFA [Text Word]) OR (quick Sequential Organ Failure Assessment* [Text Word]) OR (quick Sepsis-related Organ Failure Assessment* [Text Word]) OR (quick Sequential Sepsis-related Organ Failure Assessment* [Text Word]) AND (Systemic Inflammatory Response Syndrome [Text Word]) OR (SIRS [Text Word])) AND ((Prehospital*[Text Word]) OR (Out of hospital [Text Word]) OR (out-of-hospital [Text Word]) OR (Emergency Medical Service*[Text Word]) OR (Medical Emergency Service* [Text Word]) OR (EMS [Text Word]) OR (Ambulances[MeSH Terms]) OR (ambulance*[Text Word]) OR (paramedic*[Text Word]) OR (Emergency medical technician[MeSH Terms]) OR (emergency medical technician*[Text Word])) AND ((sepsis[Text Word]) OR (septic*[Text Word]))

**Results:** 21 search results on Jan. 10^th^, 2023, at 13:43 MEZ

## Cochrane Library: Search #2

(sepsis OR septic*):ti,ab,kw AND (Prehospital* OR (Out of hospital) OR out-of-hospital OR (Emergency Medical Service*) OR (Medical Emergency Service*) OR EMS OR ambulance* OR paramedic* OR emergency medical technician*):ti,ab,kw AND ((NEWS OR (NEWS-2) OR (National early warning score* )) AND (MEWS OR (Modified Early Warning Score*))):ti,ab,kw AND ((qSOFA OR (quick Sequential Organ Failure Assessment*) OR (quick Sepsis-related Organ Failure Assessment*) OR (quick Sequential Sepsis-related Organ Failure Assessment*)) AND ((Systemic Inflammatory Response Syndrome ) OR SIRS)):ti,ab,kw (Word variations have been searched)

**Results**: 0 search results on Jan. 10^th^, 2023, at 14:10 MEZ

# Results

After screening all identified papers (n=21), we may conclude that no study allowed any comparison of all four screening tools’ predictive ability for sepsis.
